# A Structural View of Alkyl-Coenzyme M Reductases, the First Step of Alkane Anaerobic Oxidation Catalyzed by Archaea

**DOI:** 10.1021/acs.biochem.2c00135

**Published:** 2022-05-02

**Authors:** Olivier N. Lemaire, Tristan Wagner

**Affiliations:** Max Planck Institute for Marine Microbiology, Celsiusstraße 1, 28359 Bremen, Germany

## Abstract

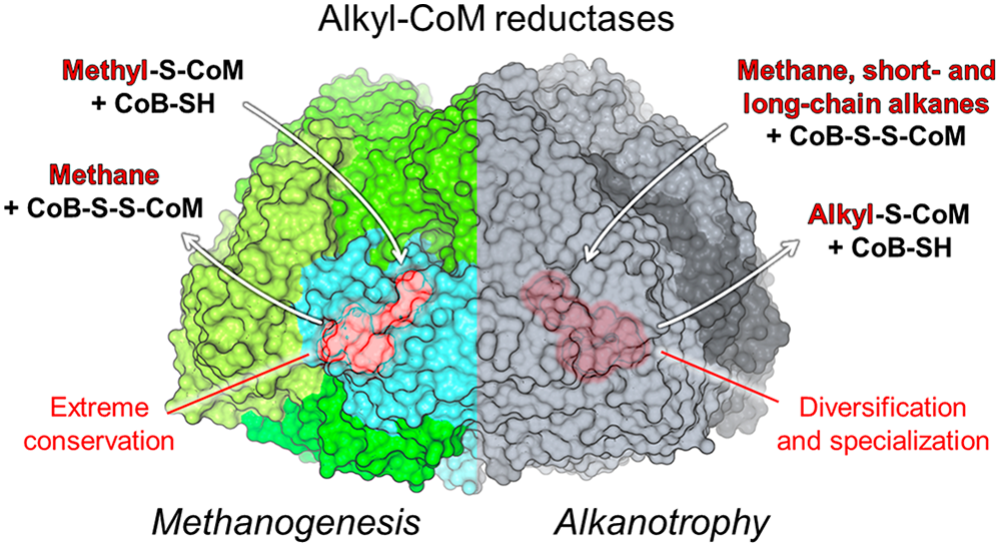

Microbial anaerobic oxidation of alkanes intrigues the scientific community by way of its impact on the global carbon cycle, and its biotechnological applications. Archaea are proposed to degrade short- and long-chain alkanes to CO_2_ by reversing methanogenesis, a theoretically reversible process. The pathway would start with alkane activation, an endergonic step catalyzed by methyl-coenzyme M reductase (MCR) homologues that would generate alkyl-thiols carried by coenzyme M. While the methane-generating MCR found in methanogens has been well characterized, the enzymatic activity of the putative alkane-fixing counterparts has not been validated so far. Such an absence of biochemical investigations contrasts with the current explosion of metagenomics data, which draws new potential alkane-oxidizing pathways in various archaeal phyla. Therefore, validating the physiological function of these putative alkane-fixing machines and investigating how their structures, catalytic mechanisms, and cofactors vary depending on the targeted alkane have become urgent needs. The first structural insights into the methane- and ethane-capturing MCRs highlighted unsuspected differences and proposed some explanations for their substrate specificity. This Perspective reviews the current physiological, biochemical, and structural knowledge of alkyl-CoM reductases and offers fresh ideas about the expected mechanistic and chemical differences among members of this broad family. We conclude with the challenges of the investigation of these particular enzymes, which might one day generate biofuels for our modern society.

Alkanes represent an efficient means of chemical energy storage, which can be released on demand for the constantly increasing energetic requirements of our modern society.^1^ They are used for heating, electricity generation, vehicle propulsion, and the synthesis of more advanced chemicals.^2^ On the contrary, these molecules exclusively composed of carbon and hydrogen are also potential pollutants. The simplest version, methane, is the greatest source of concern due to the dramatic increase in its emissions since the industrial revolution era and its greenhouse potential (84 times more than CO_2_ over a period of 20 years).^3,4^ Short-chain alkanes (ethane, propane, and butane) released into the atmosphere play a significant role in ozone formation and tropospheric photochemical pollution.^5,6^ Long-chain alkanes are also common pollutants, especially in aquatic ecosystems (e.g., oil spills^7^), and are recalcitrant to degradation.

Abiotic thermocatalytic processes naturally produce diverse alkanes that are bubbled through gas seeps with methane being the most abundant (79–85% of all hydrocarbons) followed by ethane and propane (1.38–2% and 1.25–3.1%, respectively) and butane (0.59–1.44% when combining *n*- and *iso*-butane).^8^ With regard to biological sources, methane is mainly generated by methanogenic archaea in anaerobic environments^9^ and by demethylation of methyl phosphonates in the open ocean by specific microbes, while algae, cyanobacteria, plants, and metazoans produce long-chain alkanes.^10−15^ Representing a considerable amount of carbon and cellular energy resources, these emissions are counterbalanced by numerous biological alkanotrophic processes in various environments, making alkanes key intermediates in the worldwide carbon cycle.^8,15−25^

Linear saturated alkanes (called *n*-alkanes, simplified here as alkanes) are unreactive at room temperature due to their chemical stability. Therefore, a substantial amount of energy is required to perform the homolytic cleavage of the H–C bond.^26−28^ Nevertheless, alkanes can be activated by different biological mechanisms under aerobic or anaerobic conditions. Aerobic alkanotrophic bacteria oxidize alkanes via metal-dependent alkane hydroxylases using molecular oxygen to generate the corresponding alcohols.^21,29−32^ Several families of enzymes exist within the alkane hydroxylase group, with variable specificities regarding the alkane length and their cellular localization (cytoplasmic or membrane-bound). The reaction can be performed on the terminal or penultimate (termed subterminal) carbon, and the following oxidation steps converge to the β-oxidation pathway producing acetyl-CoA, the turntable molecule of the central carbon metabolism.^21,33^

Under anaerobic conditions, microorganisms must find alternative strategies to activate alkanes. “Intra-aerobic” anaerobes such as *Candidatus* Methylomirabilis oxyfera also rely on O_2_-dependent alkane hydroxylases but generate *in cellulo* the required O_2_ by using nitrite or chlorate.^22,34−36^

Oxygen-independent alkane-activating mechanisms were also described. The most characterized anaerobic alkane oxidation metabolism is the fumarate addition.^18,37^ This “cracking” is catalyzed by the alkylsuccinate synthase (Ass/Mas) system and implies a glycyl-radical mechanism in which the activated alkane attacks fumarate to form an alkyl-succinate.^22,37,38^ The successive oxidation steps lead to the formation of acetyl-CoA and the recycling of fumarate. The electrons formed during the oxidation steps are transferred to final acceptors such as sulfate, nitrate, or ferric iron.^37^ This pathway appears to be restricted to bacteria, except for the archaeon *Archaeoglobus fulgidus* strain VC-16, which probably acquired a bacterial alkyl-succinate synthase by horizontal gene transfer.^39^ A wide range of alkanes from long-chain alkanes to propane can be oxidized.^18^ Oxidation is performed on the subterminal carbon, even if oxidation of the terminal carbon was described for propane.^40^ An ethane-dependent enrichment was cited by Kniemeyer and colleagues in 2007,^18^ but it was later shown that ethane oxidation is actually performed by archaea through another mechanism.^41^ Therefore, albeit being theoretically feasible and suggested by *in situ* measurements^19,22,42^ a fumarate-dependent activation of ethane or methane still has to be demonstrated.

Another anaerobic alkane oxidation pathway was proposed, starting with a molybdopterin-dependent alkane hydroxylase (Ahy complex) that would hydroxylate the alkane at the subterminal carbon.^37^ Such a putative mechanism is based on the activation of aromatic alkanes by the ethylbenzene dehydrogenase and would explain how strictly anaerobic sulfate-reducing bacteria lacking the alkylsuccinate synthase, such as *Desulfococcus oleovorans* Hxd3, can grow on alkanes.^37,43−46^ The hydroxylated product would be further oxidized to a ketone, followed by a carboxylation at the C3 position, which would allow the formation of a CoA ester.

The last anaerobic alkane oxidation strategy known to date has been reported only in archaea and involves the formation of a thiol adduct that is dependent on a radical mechanism. The reaction is assumed to be catalyzed by the methyl-coenzyme M reductase (MCR) family harboring a nickel-containing porphinoid, the cofactor F_430_ (Figure 1^47−51^).

**Figure 1:**
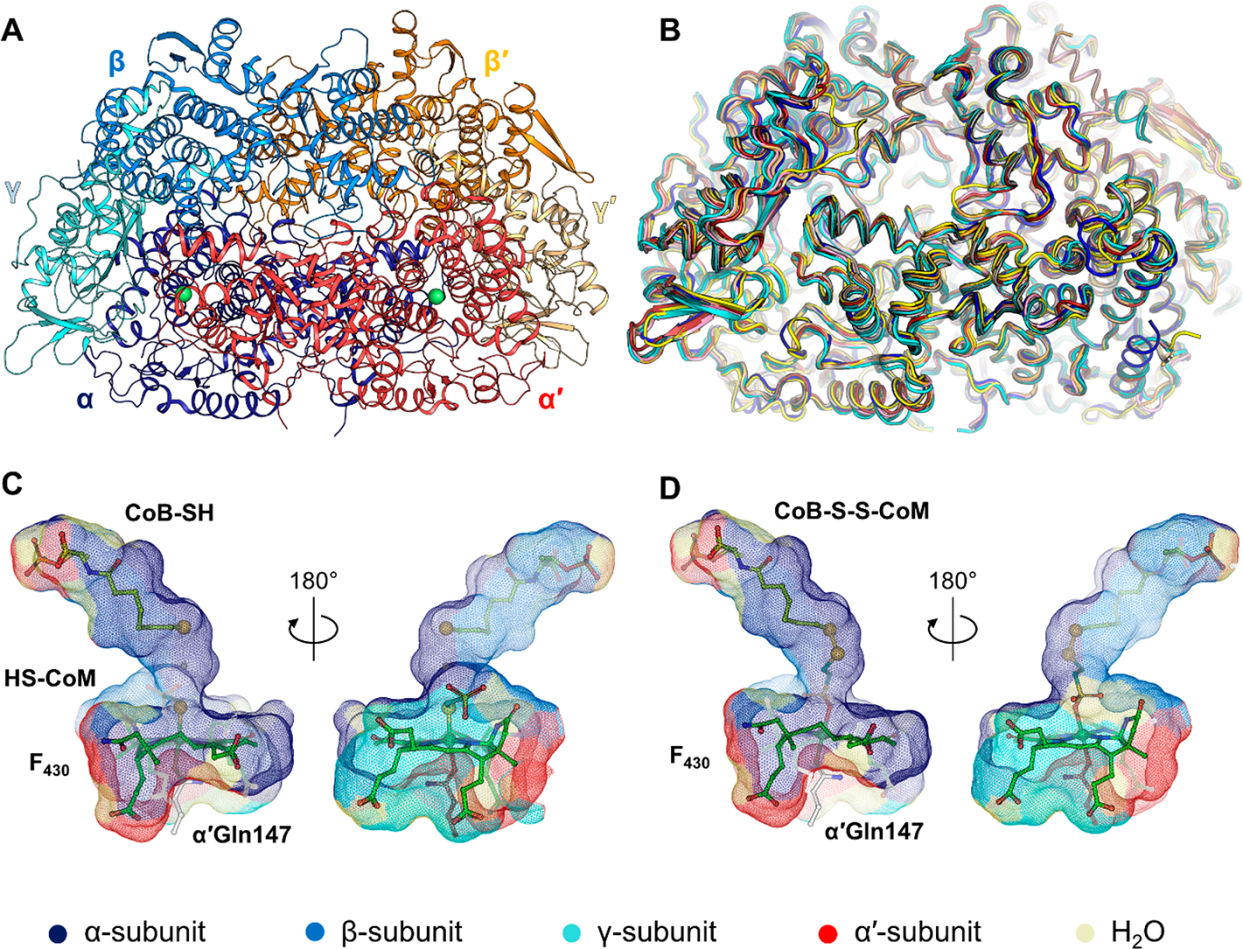
Anatomy of a canonical MCR and structural conservation across the methanogenic enzymes. (A) Structure of a canonical αα′ββ′γγ′ heterohexameric MCR. The structure (*Methanothermobacter marburgensis* type I MCR, PDB entry 5A0Y) is represented by a cartoon, and each chain is colored differently. The position of the catalytic nickel is indicated by a green sphere. (B) Superposition of nine MCR structures from different methanogenic archaea (PDB entry 1E6V from *Methanopyrus kandleri* in dark red, PDB entry 1E6Y from *Methanosarcina barkeri* in dark blue, PDB entry 5A0Y from *M. marburgensis* MCR type I in white, PDB entry 5A8K from *Methanothermobacter wolfeii* MCR type I in wheat, PDB entry 5A8R from *M. marburgensis* MCR type II in light pink, PDB entry 5A8W from *M. wolfeii* MCR type II in light orange, PDB entry 5N1Q from *Methanothermococcus thermolithotrophicus* in cyan, PDB entry 5N28 from *Methanotorris formicicus* in teal, and PDB entry 7NKG from *Methermicoccus shengliensis* in yellow). (C) Position of reduced coenzymes in the active site of MCR (PDB entry 5A0Y). (D) Position of oxidized coenzymes in the active site of MCR (PDB entry 1HBM). In panels C and D, binding sites are contoured by a transparent surface and colored according to the involved subunit or element as indicated. F_430_, CoB-SH, HS-CoM, and α′Gln147 are shown as sticks and colored green, pale green, cyan, and white, respectively. Oxygen, nitrogen, sulfur, phosphate, and nickel are colored red, blue, yellow, orange, and green, respectively. Catalytic nickel and the reactive sulfur atoms of coenzymes are represented as spheres.

Initially proposed for methanotrophic archaea, the enzyme and the corresponding alkyl-thiols have been detected in ethane, propane, butane, and even long-chain hydrocarbon anaerobic oxidizers.^19,20,25,41,52−56^ Such metabolic processes play a fundamental role in the carbon cycle and could have considerable impacts on the development of new strategies for alkane mitigation. Because the overall reaction is close to equilibrium, these enzymes could also be tamed for biological alkane production.

This Perspective aims not to be another review of the methanogenic MCR and its reaction, already recently described,^47,57−60^ nor a compilation of information about the phylogeny and ecological aspects of alkane oxidizers and their homologues of MCR, also recently reviewed.^23,40,61^ It offers a different angle of reflection on the alkyl-coenzyme M reductases (ACRs) based on the recently performed biochemical and structural investigations. After a short summary of the knowledge gathered on these enzymes and the organisms that produce them, we will discuss the similarities and particularities among the different ACRs and how they could impact their specificities and reaction paths. Finally, we will explain the challenge of studying them and their possible application.

## Discovery and Diversity of Coenzyme M-Dependent Alkanotrophic Archaea

Anaerobic methanotrophic archaea (in short ANME for anaerobic methanotroph) consume more than 80% of the methane produced in the oceans where they can organize in gigantic microbial mats settled on hundreds of active gas seeps.^16,17^ It has been proposed that this astounding methane sink played a crucial role in methane cycling during the Archean period before oxygen became available for abiotic and biological methane oxidation.^17,62^ The spatial distribution of ANMEs is, however, not limited to these specific ecological niches and is broader than previously thought.^15,61,63−68^

The archaea oxidize methane into CO_2_, and the generated reducing equivalents are transferred to different final electron acceptors: nitrate, Fe(III), or Mn(IV) for the ANME-2d group (*Candidatus* Methanoperedens, family Methanoperedenaceae)^69−74^ and sulfate-reducing bacteria for other ANMEs.^61,75^ The electron transfer from the archaea to the partner bacteria is still not completely understood, but a direct interspecies electron exchange by nanowires is the most commonly proposed scenario.^61,63,76,77^ The overall process is exergonic and allows the archaea and bacteria to derive their cellular energy (Table 1).

**Table 1:** Thermodynamics of Methanogenesis and Alkanotrophy^a^

metabolism	reaction	standard Gibbs free energy change	ACR direction and substrate	ref
Hydrogenotrophic methanogenesis	4H_2_ + CO_2_ → **CH**_**4**_ + 2H_2_O	–131 kJ mol^–1^ of CH_4_	CH_3_-S-CoM + HS-CoB → **CH**_**4**_ + CoB-S-S-CoM	(127)
Methylotrophic methanogenesis	4CH_3_OH → **3CH**_**4**_ + CO_2_ + 2H_2_O	–106.5 kJ mol^–1^ of CH_4_	CH_3_-S-CoM + HS-CoB → **CH**_**4**_ + CoB-S-S-CoM	(127)
Aceticlastic methanogenesis	CH_3_COO^–^ + H^+^ → **CH_4_** + CO_2_	–36 kJ mol^–1^ of CH_4_	CH_3_-S-CoM + HS-CoB → **CH**_**4**_ + CoB-S-S-CoM	(127)
Anaerobic methane oxidation coupled to SO_4_^2–^ reduction	**CH**_**4**_ + SO_4_^2–^ + 2H^+^ → CO_2_ + H_2_S + 2H_2_O	–21 kJ mol^–1^ of CH_4_	**CH**_**4**_ + CoB-S-S-CoM → CH_3_-S-CoM + HS-CoB	(128)
Anaerobic methane oxidation coupled to NO_3_^–^ reduction	**CH**_**4**_ + 4NO_3_^–^ → CO_2_ + 4NO_2_^–^ + 2H_2_O	–521 kJ mol^–1^ of CH_4_	**CH**_**4**_ + CoB-S-S-CoM → CH_3_-S-CoM + HS-CoB	(128)
Anaerobic methane oxidation coupled to MnO_2_ reduction	**CH**_**4**_ + 4MnO_2_ + 8H^+^ → CO_2_ + 4Mn^2+^ + 6H_2_O	–763.2 kJ mol^–1^ of CH_4_	**CH**_**4**_ + CoB-S-S-CoM → CH_3_-S-CoM + HS-CoB	(61)
Anaerobic methane oxidation coupled to Fe^3+^ reduction	**CH**_**4**_ + 8Fe^3+^ + 2H_2_O → CO_2_ + 8Fe^2+^ + 8H^+^	–454 kJ mol^–1^ of CH_4_	**CH**_**4**_ + CoB-S-S-CoM → CH_3_-S-CoM + HS-CoB	(72)
Anaerobic ethane oxidation coupled to SO_4_^2–^ reduction	**4C**_**2**_**H**_**6**_ + 7SO_4_^2–^ + 14H^+^ → 8CO_2_ + 7H_2_S + 12H_2_O	–73.2 kJ mol^–1^ of C_2_H_6_	**C**_**2**_**H**_**6**_ + CoB-S-S-CoM → C_2_H_5_-S-CoM + HS-CoB	(41)
Anaerobic propane oxidation coupled to SO_4_^2–^ reduction	**2C**_**3**_**H**_**8**_ + 5SO_4_^2–^ + 4H^+^ → 6HCO_3_^–^ + 5H_2_S + 2H_2_O	–102 kJ mol^–1^ of C_3_H_8_	**C**_**3**_**H**_**8**_ + CoB-S-S-CoM → C_3_H_7_-S-CoM + HS-CoB	(18)
Anaerobic butane oxidation coupled to SO_4_^2–^ reduction	**4C**_**4**_**H**_**10**_ + 13SO_4_^2–^ + 10H^+^ → 16HCO_3_^–^ + 13H_2_S + 4H_2_O	–138 kJ mol^–1^ of C_4_H_10_	**C**_**4**_**H**_**10**_ + CoB-S-S-CoM → C_4_H_9_-S-CoM + HS-CoB	(18)
Anaerobic hexadecane oxidation coupled to methanogenesis	**4C**_**16**_**H**_**34**_ + 30H_2_O → 49CH_4_ + 15CO_2_	–339.2 kJ mol^–1^ of C_16_H_34_	**C**_**16**_**H**_**34**_ + CoB-S-S-CoM → C_16_H_33_-S-CoM + HS-CoB	(54)

a The metabolisms proposed for methanogens^127^ and anaerobic alkane oxidizers^18,41,54,61,72,128^ are indicated, as well as the associated Gibbs free energy changes and the reactions performed by the ACRs.

The methane activation reaction is considered to be a reversal of methane formation during the final step of methanogenesis. According to this scenario, a radical mechanism involving the heterodisulfide made of coenzymes M and B (CoB-S-S-CoM) would react with methane, generating methyl-S-CoM and HS-CoB.^47,57,59^ The overall reaction would be catalyzed by the Ni^I^-cofactor F_430_ deeply buried in the MCR. This activity has never yet been confirmed in an enzyme isolated from an ANME. It is nevertheless largely accepted by the scientific community as supported by indirect evidence. (1) The three subunits composing MCR were found in a large amount at the protein and transcript levels in methane-oxidizing mats and enrichments.^72,74,78,79^ (2) The addition of the specific MCR inhibitor bromoethanesulfonate (BES) abolishes methane uptake in some ANMEs.^80^ (3) The MCR from methanogens can generate methyl-S-CoM from methane and the heterodisulfide *in vitro* and *in vivo*.^81,82^ (4) Heterologous expression of the MCR from an ANME in the methanogen *Methanosarcina acetivorans* led to an increased methane oxidation rate.^82,83^ In the latter case, it is, however, unclear whether overexpression of the native MCR from *M. acetivorans* would not lead to the same phenotype as the production of the MCR from ANME. In 2011, a fascinating structure of the MCR from an ANME-1 was literally raised from the sea: the protein used for X-ray crystallography was directly purified from Black Sea methane-oxidizing microbial mats.^84^ This structure is highly similar to that of the MCR used by methanogens and contains both bound reduced coenzymes, corroborating that MCR should be the key actor of the methane sink.

**Figure 2:**
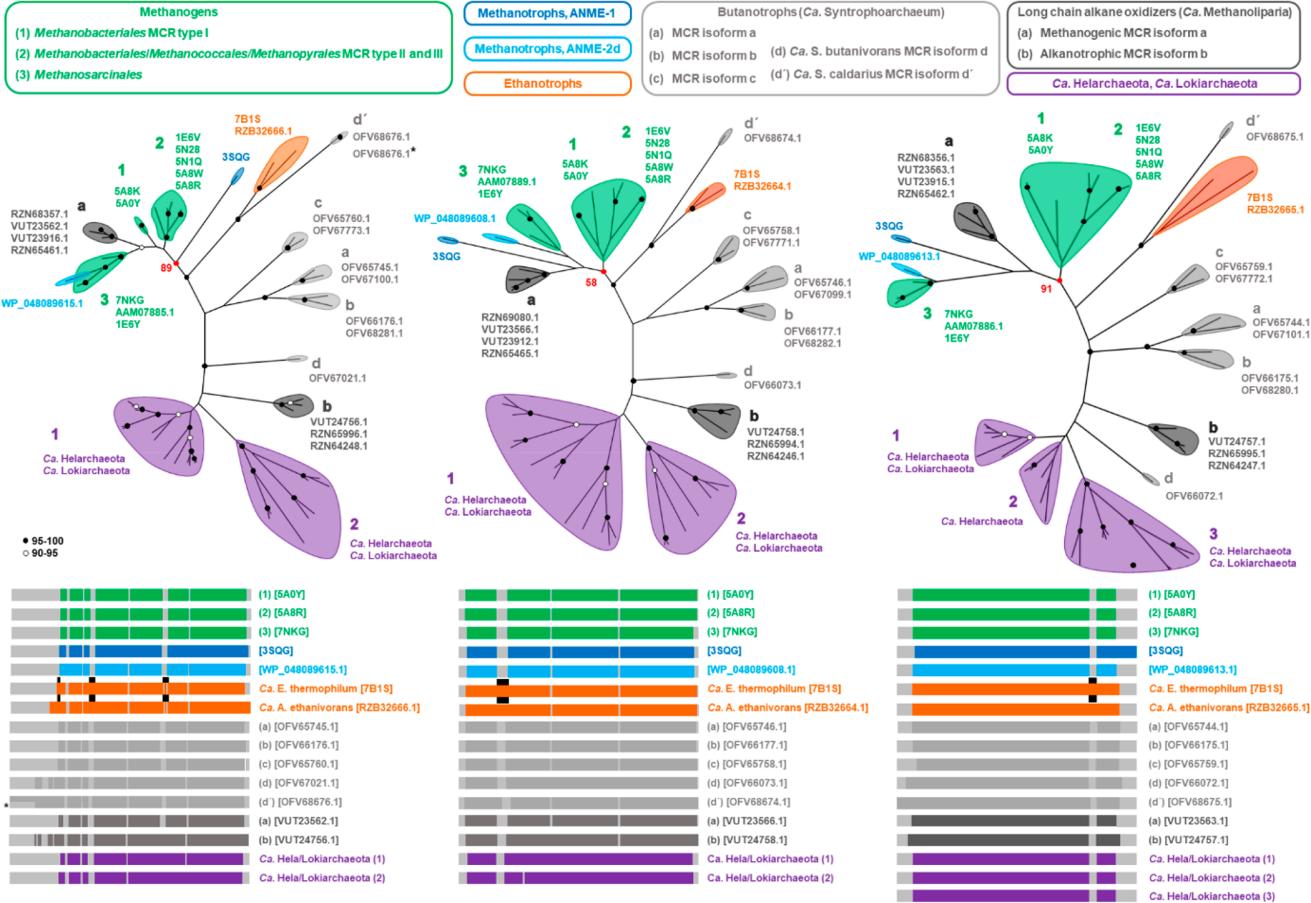
Phylogeny distribution and differences in ACR sequences. Phylogenetic tree of McrA (left), McrB (middle), and McrG (right). Sequences from methanogens and alkanotrophs are colored as indicated in the top and bottom panels. The accession number of the used sequences is indicated. The trees were constructed using the maximum likelihood method by the MEGA-X program.^129^ Nodes with a bootstrap superior to 90 (500 replications) are represented. The node of the branch containing all methanogenic and methanotrophic MCRs is colored red. Aligned sequences and their respective accession numbers are represented in the bottom panel and colored according to the top panel. A light gray color indicates an area of insertion. Black blocks highlight the insertions involved in tunnel entry in the enzyme from *Ca*. E. thermophilum (PDB entry 7B1S). Because of a suspicious N-terminal extension, the OFV68676.1 sequence was used as complete or truncated (marked with an asterisk) for the elaboration of the figure. All sequences used for the *Ca*. Helarchaeota and *Ca*. Lokiarchaeota were obtained from the transcriptome shotgun assembly database. AAM07885.1, AAM07889.1, and AAM07886.1 come from *M. acetivorans* strain C2A.

Due to the complexity and the singularity of the reaction, the thiol adduct chemistry was assumed to occur for only methane. However, in 2016, a deep-sea microbial consortium broke the dogma: the HS-CoM-dependent oxidation of propane and butane was demonstrated.^52^ From alkane-rich seep sediments, Laso-Pérez and colleagues obtained a butane-oxidizing consortium in which the archaeal population was dominated by *Candidatus* Syntrophoarchaeum butanivorans and *Candidatus* Syntrophoarchaeum caldarius belonging to the *Methanosarcinales* order. The culture completely oxidized butane to CO_2_ with the concomitant reduction of sulfate by a bacterial partner. The addition of BES to the culture inhibited butane degradation. The genome of both archaea encodes four divergent MCR homologues (Figure 2), and transcriptomics/proteomics showed high levels of abundance for most of them. A propane-dependent sulfate reduction was also described after a two-month incubation of this enrichment, albeit modifications of microbial composition and transcriptomics/proteomics experiments were not assessed in this culture. Propyl-S-CoM and butyl-S-CoM were detected upon the addition of the respective alkanes (propyl-S-CoM being detected by its expected size, without a standard). The consortium, however, appeared to be unable to oxidize smaller or larger alkanes (e.g., methane, ethane, pentane, or hexane). Altogether, these data support the hypothesis that *Ca*. S. butanivorans and *Ca.* S. caldarius are butane and propane oxidizers and that at least one of their MCR homologues is used to activate the alkanes.

With this breakthrough, the hunt for archaea that can oxidize non-methane alkanes began.

In 2019 and 2020, two consecutive works reported the enrichment of ethane-oxidizing consortia living under psychrophilic and thermophilic conditions, respectively.^41,53^ Similarly to butane, the alkane oxidation was shown to be dependent on sulfate reduction from a bacterial partner (Table 1), and ethyl-S-CoM was detected upon addition of ethane to both cultures. The psychrophilic culture was dominated by *Candidatus* Argoarchaeum ethanivorans, and the thermophilic culture by *Candidatus* Ethanoperedens thermophilum. Both archaeal genomes encode a single MCR homologue that is abundantly produced. Further work performed by Hahn and colleagues on the thermophilic consortium showed that ethyl-S-CoM was the only alkyl-S-CoM detected upon addition of a mixture composed of methane, ethane, propane, and butane and that ethane oxidation was inhibited by BES.^53,85^ The structure of the MCR homologue of the proposed ethanotroph *Ca*. E. thermophilum was obtained only a year later, highlighting a similar architecture with unsuspected structural traits described below.^85^

Long-chain alkane degradation by a novel archaeal lineage, *Candidatus* Methanoliparia, was first suspected on the basis of metagenome-assembled genomes (MAGs) obtained from oil-rich samples.^54,55^*Ca*. Methanoliparia species were proposed to use a divergent MCR homologue to activate mid- to long-chain alkanes as alkyl-S-CoM and ultimately release CO_2_ and methane (Table 1 and Figure 2). Methane would be generated by the second isoform of MCR encoded in each MAG and evolutionarily closer to the MCRs found in methanogens (Figure 2). Two years later, the study of an oil-degrading consortium forming CO_2_ and methane and rich in *Ca*. Methanoliparia confirmed some of these hypotheses.^25^ The addition of various alkanes such as tetradecane (C14), pentadecane (C15), hexadecane (C16), eicosane (C20), docosane (C22), and nonlinear alkanes such as hexadecylcyclohexane and hexadecylbenzene led to methane generation and the detection of the respective alkyl-S-CoM (only hexadecyl-S-CoM and eicosyl-S-CoM standards were used; others were identified by their theoretical masses). The enrichment appeared to be unable to oxidize smaller alkanes (C2–C8). Assuming that *Ca*. Methanoliparia species are responsible for the alkane oxidation and their divergent MCR homologue catalyzes the first step, these results raise questions about how such an enzyme would recognize a wide range of (non)-linear long-chain alkanes.

Recent metagenomic studies detected genes encoding proteins homologous to those involved in methanogenesis and alkanotrophy, including MCR homologues, in MAGs of uncultured archaea from the *Candidatus* Bathyarchaeota, Helarchaeota, and Lokiarchaeota phyla.^15,86−89^ This appears to contradict the previous hypothesis that methanogenesis and by extension the HS-CoM-dependent alkanotrophy appeared early in the evolution of *Euryarchaeota*.^90^ It has been concluded that organisms from the *Ca*. Bathyarchaeota phylum contain an incomplete methanogenesis/alkanotrophic pathway while the genome of some *Ca*. Helarchaeota species encodes the complete set of enzymes allowing these archaea to perform methanogenesis or alkanotrophy under some conditions.^86−89,91^ The alkane oxidation would rely on an association with sulfate-reducing bacteria.^92^

In the mentioned methanogens and alkanotrophs, the alkane chemistry is supposed to be performed by ACRs. But is the phylogeny of these ACRs distributed according to the organisms carrying them, and do they share similar structural and mechanistic features?

## Overall Organization and Phylogeny Distribution of ACRs

To avoid confusion, the following nomenclature will be used to describe ACRs: methanogenic MCRs for the enzymes involved in the methanogenesis process, generating methane from methyl-S-CoM and CoB-SH; methanotrophic MCRs for the enzymes encoded in ANME genomes proposed to capture methane with the heterodisulfide; and MCR homologues for the enzymes found in organisms degrading longer alkanes. All of these enzymes are gathered as ACRs. It is worth noting that without any enzymatic characterization of these proteins, such appellations are still speculative except for the methanogenic one. Similarly, in the absence of a pure culture, the proposed alkanotrophic archaea have not been demonstrated to perform the respective alkane oxidation but they will be considered as such in the text for the sake of clarity.

**Figure 3:**
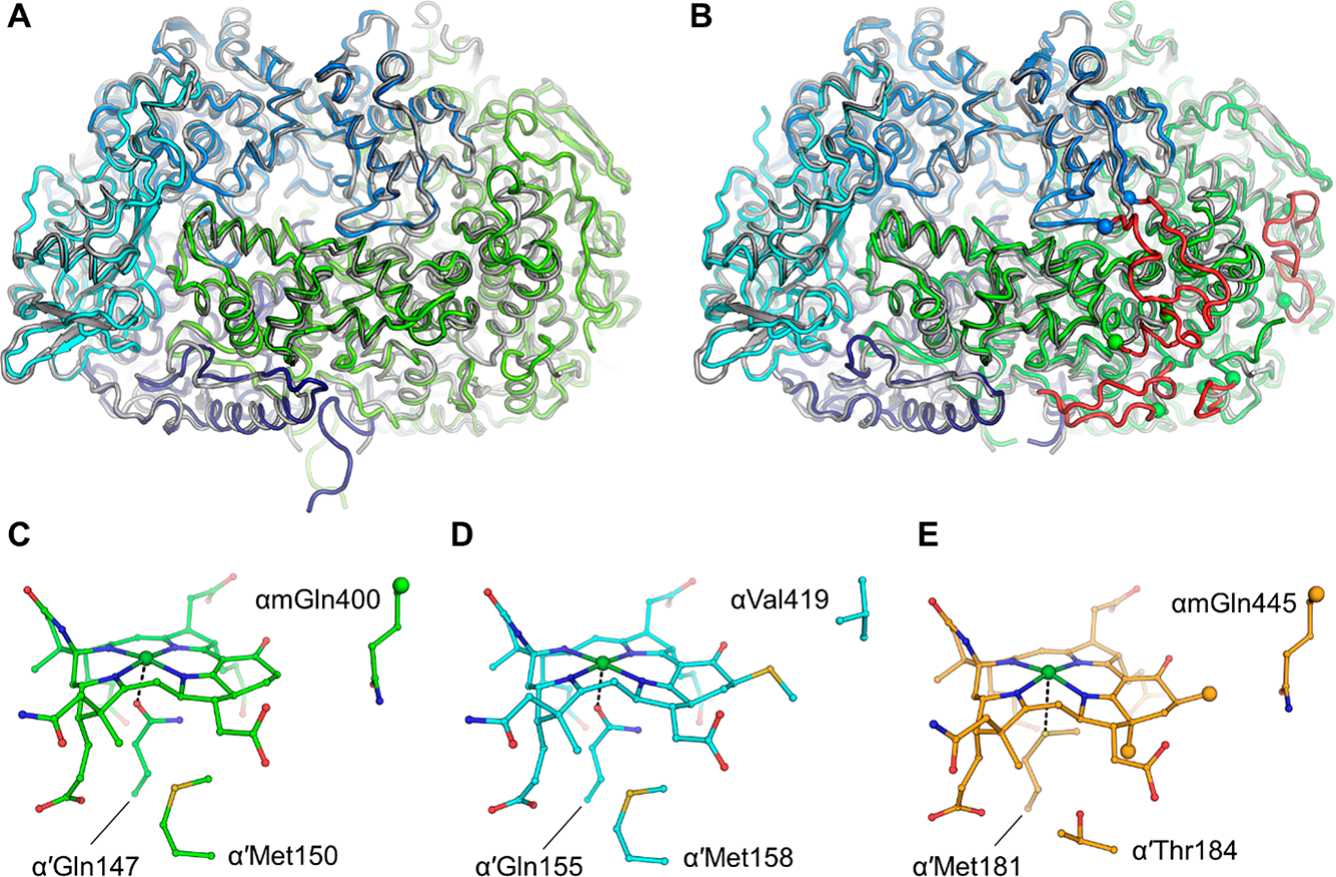
Differences between methanogenic and methanotrophic MCRs, and the MCR homologue from *Ca*. E. thermophilum. (A and B) Structural superposition of the structures from methanogenic MCR type I from *M. marburgensis* (PDB entry 5A0Y) with Black Sea mat methanotrophic MCR (PDB entry 3SQG, A) or the MCR homologue from *Ca*. E. thermophilum (PDB entry 7B1S, B). In both panels, methanogenic MCR is colored gray and methanotrophic MCR or the homologue from *Ca*. E. thermophilum is colored by chain (α in dark blue, β in blue, γ in cyan, and α′β′γ′ in green). The additional loops of the α′βγ′ subunits are colored red, and the insertion sites are indicated by sphere for *Ca*. E. thermophilum. (C–E) F_430_ cofactors and varying surrounding residues from methanogenic MCR (PDB entry 5A0Y, C, green), methanotrophic MCR (PDB entry 3SQG, D, cyan), and the MCR homologue from *Ca*. E. thermophilum (PDB entry 7B1S, E, orange). A dashed line links the catalytic nickel (green sphere) and its lower axial ligand. Additional methylations are represented by spheres. Oxygen, nitrogen, and sulfur are colored red, blue, and yellow, respectively.

All characterized ACRs form a compact heterohexameric structure made of an α_2_β_2_γ_2_ architecture (Figure 1A) and harbor a nickel-containing porphinoid named cofactor F_430_, due to its absorption at this wavelength.^85,93^ The X-ray crystal structures of nine methanogenic MCRs from various methanogens are accessible in the Protein Data Bank (PDB). They show a remarkable three-dimensional superposition and a perfectly conserved active site (Figure 1B^85^). The binding sites of the F_430_ cofactor and the coenzymes are at the interface of the αα′βγ subunits [the prime indicates the homodimeric subunits (Figure 1C,D)]. In methanotrophs, the structure of MCR obtained from Black Sea mats (ANME-1) presents the typical hexameric organization [282.5 kDa (Figure 3A)], but a MCR complex of ≈810 kDa proposed to form trimeric α_2_β_2_γ_2_-hexamers was interestingly reported in *Ca*. Methanoperedens BLZ2 (ANME-2d clade).^94^ Because *Ca*. Methanoperedens BLZ2 is mesophilic (30 °C), this higher degree of organization would be unlikely to be used for thermo-tolerance and might instead be used for regulation or to contact different cellular partners. It is worth noting here that differences among the methanotrophic MCRs are expected, as ANMEs make up a polyphyletic group and exhibit divergent MCRs (Figure 2). The last ACR structurally characterized to date is the MCR homologue from the ethanotroph *Ca*. E. thermophilum.^85^ This ACR is still hexameric but differs in length from all other characterized MCR homologues by insertions in the three subunits (Figures 2 and 3B). These insertions are specific to MCR homologues from ethanotrophs (Figure 2) and are thought to be crucial for alkane selectivity, as discussed below.

The phylogenetic tree of ACRs indicates a relatively clear separation between methanogenic/methanotrophic MCRs (including the proposed methanogenic MCR from *Ca*. Methanoliparia) and the MCR homologues from alkane oxidizers (Figure 2). Until recently, the knowledge gathered on ACRs was restricted to only a subgroup of these enzymes. Assuming a standard genetic code, the ACRs that would be produced in *Ca*. Helarchaeota and *Ca*. Lokiarchaeota branch with the MCR homologues from *Ca*. Methanoliparia species, regardless of the subunit used for tree generation and as previously published (Figure 2^91^). However, unlike *Ca*. Methanoliparia species, no other sequences branching with methanogenic MCRs have been found in *Ca*. Helarchaeota or *Ca*. Lokiarchaeota groups, suggesting that a putative alkane oxidation process in these organisms would release only CO_2_ without the formation of methane.

Sequences from MCR homologues are mostly derived from automatic annotation, and only a few were experimentally verified. The determination of the initiating codon may be incorrect for some sequences. For instance, α subunits of MCR homologues from *Ca*. S. caldarius (OFV68676.1) and *Ca*. A. ethanivorans (RZB32666.1) exhibit a suspicious N-terminal extension of 86 and 36 residues, respectively, before the next methionine (Figure 2). These extensions could come from a wrong annotation of the initial methionine, and mass spectrometry data or a biochemical/structural characterization must confirm if these extensions actually exist. Such artifacts do not impact the obtained phylogenetic tree of the MCR homologues (see OFV68676.1 in Figure 2), but the role of such unverified extensions will not be discussed herein.

## Differences in Cofactors and Post-translational Modifications

It is assumed that all ACRs require the F_430_ cofactor as a catalyst. While all characterized methanogenic MCRs harbor the classical F_430_ in their active site, methanotrophic MCRs from the ANME-1 cluster contain a modified methylthio-F_430_ (Figure 3C,D^79,84,95,96^). The function and biosynthesis of this methylthio-group addition are still elusive, but it appears not to be strictly necessary for methane fixation because a classic F_430_ was found in MCR from the ANME-2 clade.^96^ The enzyme purified from the ethanotroph *Ca*. E. thermophilum contained another variation of the cofactor, a dimethyl-F_430_, as shown by structural data and mass spectrometry (Figure 3E^85^). The methylations of the cofactor have been proposed to be installed by a methyl-transferase belonging to the CobM2 family, encoded in the genome of ethane oxidizers.^85^ These methylations do not modify the optical properties of the cofactor extracted from the protein and have been proposed to serve as anchors to maintain its integrity and lock its correct position in the catalytic chamber, which is wider than those of methanogenic and methanotrophic MCRs.

**Figure 4:**
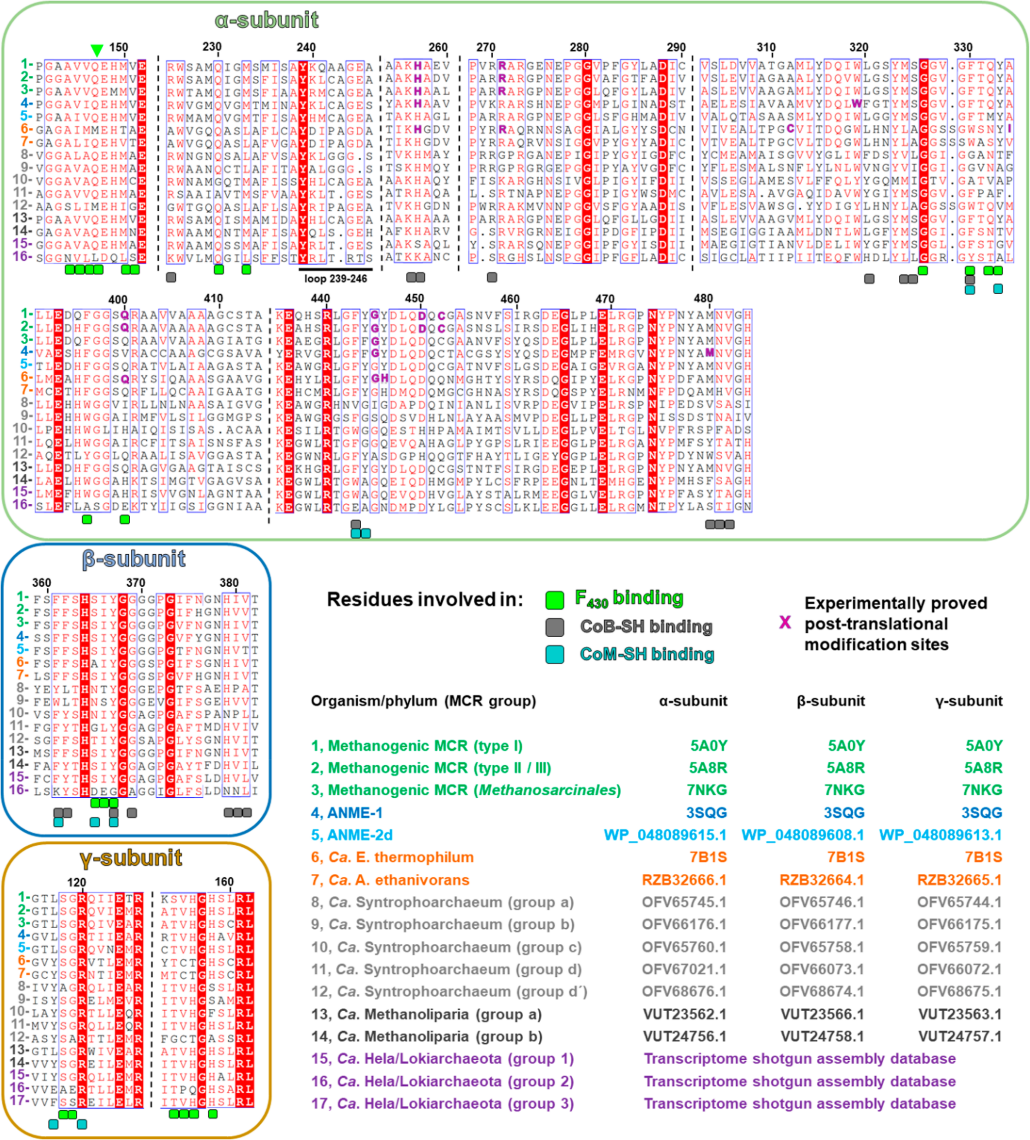
Alignment of ACRs and residues involved in the stabilization of coenzymes and cofactors. The residues involved in the coordination of F_430_, HS-CoB, and HS-CoM were taken from ref (85). The numbering is based on the structure of MCR type I from *M. marburgensis* (PDB entry 5A0Y). The aligned sequences are colored according to the organism and its proposed metabolism as in Figure 2. Post-translational modifications experimentally proven are indicated (see also Figure 5). A green arrow indicates the position of the lower axial ligand of the catalytic nickel. The alignment was performed with Clustal Omega^130^ with default parameters, and the figure was constructed with Espript.^131^

It is interesting to note that F_430_ modifications can be correlated with some specific residue substitutions. For example, the structure of the MCR from an ANME-1 showed that the methylthio-group addition is accommodated by substitution of the methyl-glutamine (residue α400 in *M. marburgensis* MCR type I) with a valine (Figures 3C,D and 4(84)). Such a small hydrophobic residue (valine or alanine) is found at this position in McrA sequences from the ANME-1 cluster, but not in those from the ANME-2 cluster. The presence of a methylthio-F_430_ could therefore be hypothesized on the basis of the McrA sequence. The two methylations carried by the cofactor of the MCR homologue from *Ca*. E. thermophilum (Figure 3E^85^) are not bulky enough to induce clashes with the methyl-glutamine, unlike the methylthio-group addition. The (methyl-)glutamine is conserved in the MCR homologues from *Ca*. A. ethanivorans, one of the MCR homologues from *Ca*. S. caldarius (OFV68676.1), and the putative methanogenic MCRs from *Ca*. Methanoliparia. However, this position is intriguingly substituted with isoleucine, histidine, or glutamate in other MCR homologues (Figure 4), raising questions about the potential modifications that may be harbored at this F_430_ position.

Another interesting substitution is the axial ligand coordinating the catalytic nickel of the F_430_ cofactor, for which the canonical glutamine is replaced by methionine in the enzyme from *Ca*. E. thermophilum.^85^ Such a substitution, also found in a MCR homologue from *Ca*. S. caldarius (OFV68676.1), is not a key functional element because other ethanotrophs such as *Ca*. A. ethanivorans contain the canonical glutamine at this position (Figure 4). The F_430_–methionine interaction might impact the electron repartition in the porphinoid or tune the nickel oxido-reduction potential.^60^ Some sequences from *Ca*. Helarchaeota and Lokiarchaeota species interestingly exhibit a leucine at this position that might also confer additional reactivity properties (Figure 4).

While methanogenic and methanotrophic MCRs share an almost identical binding interface for CoM-SH and CoB-SH (Figure 4), few substitutions appear in MCR homologues from ethanotrophs, which slightly rearranged the position of coenzymes as seen in the structures.^85^ More substitutions exist in MCR homologues from *Ca*. Syntrophoarcheum and *Ca*. Methanoliparia, contrasting with the strict conservation in methanogenic/methanotrophic MCRs (Figure 4). A good example is the α480 methionine (*M. marburgensis* numbering) in the vicinity of the CoB-SH position that is substituted with a tryptophan in the MCR homologue from *Ca.* S. caldarius [OFV68676.1 (Figure 4)]. The sequences from *Ca*. Helarchaeota and Lokiarchaeota exhibit even more drastic substitutions in residues involved in the coordination of cofactor and coenzymes despite a relatively good degree of residue conservation with methanogenic MCRs [∼40% identity with the *M. marburgensis* type I MCR (Figure 4)]. Without compensation, these drastic substitutions would modify the environment of the cofactor and coenzymes or a variation of these molecules. All of these uncharacterized ACRs could therefore hide some interesting and new features that could be assessed by structural analysis, mass spectrometry, or physiological experiments using coenzyme-mimicking molecules (e.g., BES) as culture-specific inhibitors.

**Figure 5:**
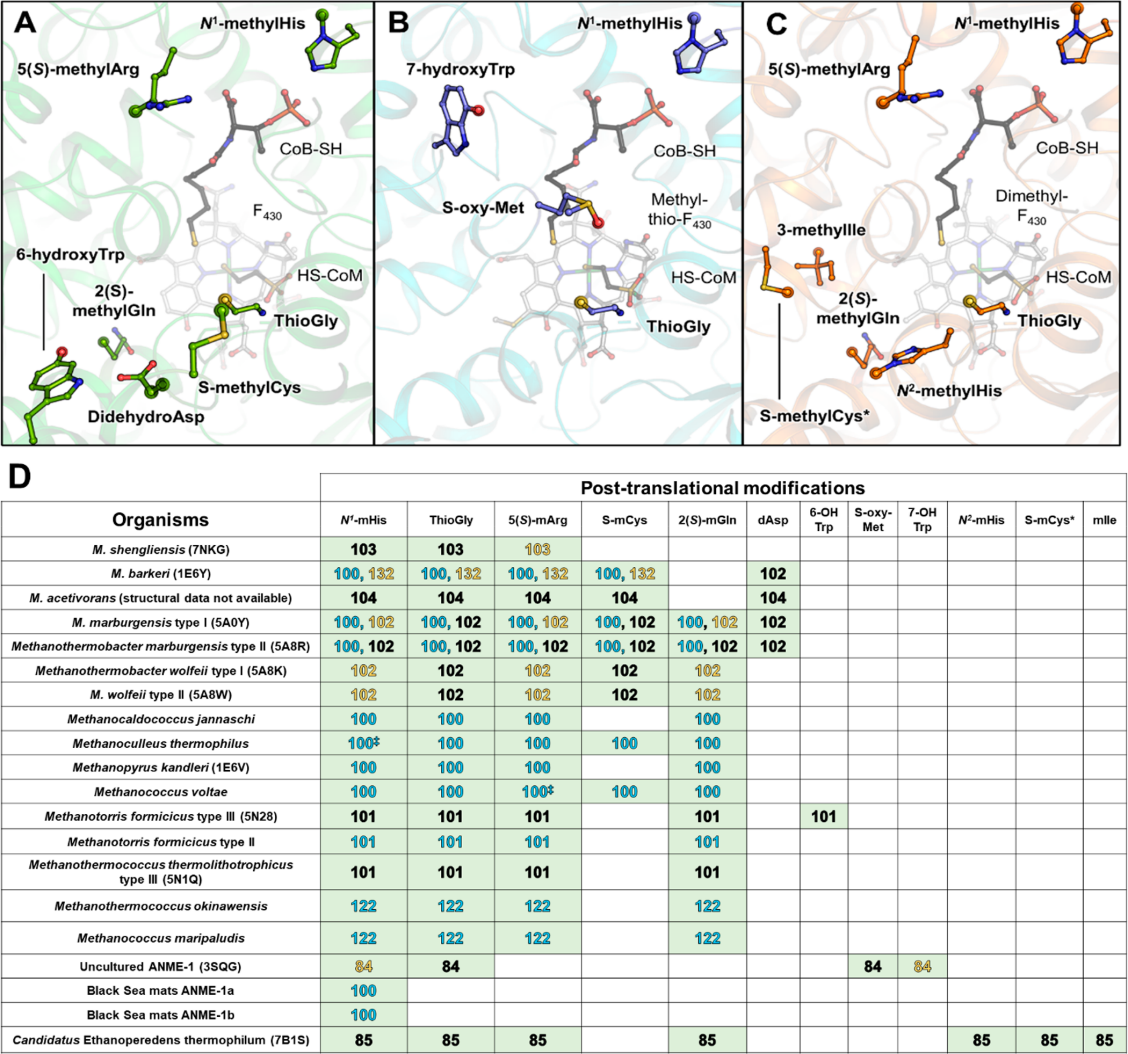
Post-translational modifications in characterized ACRs. (A–C) Positions of the post-translational modification sites in methanogenic MCR (PDB entry 5A0Y, green), methanotrophic MCR (PDB entry 3SQG, navy), and the MCR homologue from *Ca*. E. thermophilum (PDB entry 7B1S, orange), respectively. The 6-hydroxy-tryptophan found in *M. formicicus* has been superposed and added to panel A but does not exist in the original model from *M. marburgensis*. Proteins are represented as cartoons with the cofactor, coenzymes, and modified residues shown as balls and sticks. Cofactors F_430_ are colored white, and coenzymes black. Oxygen, nitrogen, sulfur, phosphate, and nickel are colored red, blue, yellow, orange, and green, respectively. Residue modifications are represented by transparent spheres. (D) Experimentally demonstrated post-translational modifications in characterized ACRs. Modifications demonstrated by X-ray crystallography, mass spectrometry, or both methods are colored yellow, blue, or black, respectively, with the corresponding reference.^84,85,100−104,122,132^ A double dagger indicates the modifications that are supposed to be present even if the peptides were not detected by mass spectrometry.^100^

The several post-translational modifications decorating the α subunit are also a unique and interesting feature of MCRs^97−99^ (Figures 4 and 5). Because such modifications are mainly localized in the vicinity of the active site, their presence is variable, and they can potentially be interexchangeable. For instance, the MCR from ANME-1 lacks the methylation on the arginine [position α285 in 3SQG (Figures 4 and 5)^84,100^], but the hydroxyl group harbored by a 7-hydroxy-tryptophan (position α333 in 3SQG) occupies a similar position and appears to compensate for the loss.^84^ A similar hypothesis was emitted in the methanogenic MCR from *Methanotorris formicicus*, in which the 6-hydroxy-tryptophan (position α429) was suggested to functionally compensate for the lack of didehydroaspartate found in some methanogenic MCRs (Figure 5^101,102^).

The physiological functions of such modifications have not yet been decrypted. A minimum set of three modifications was observed in *M. shengliensis*; two of them, the 5(*S*)-methylarginine and thioglycine, are not essential as shown by genetics, even if their absence could have dramatic effects on growth depending on the supplied substrate, culture temperature, and methane production.^99,103−105^ Structural superposition shows that additional modifications have only a small, even negligible, impact on the local environment. Of course, it must be mentioned here that all of these MCR structures used for comparison are crystallographic snapshots, with most of them in the inactive state (Ni^II^-silent). The modifications might have unexpected impacts during catalytic turnover, exemplified by the proposed role of the thioglycine as a noncrucial but activity-improving stabilizing agent of the active site surroundings.^99^ They might also have a role in the correct MCR assembly or confer advantageous properties under specific physiological conditions.^104,105^

The structure of the MCR homologue from *Ca*. E. thermophilum, harboring an extra set of post-translational modifications, spawned some fresh hypotheses regarding their potential functions (Figures 4 and 5(85)). While four of them are common to other MCRs, three specific modifications (methylisoleucine, *N*^2^-methylhistidine, and methylcysteine) are spatially clustered together to maintain a hydrophobic open ring close to the active site. Such architecture maintains an internal hydrophobic cavity that should enhance the rate of transfer of ethane to the catalytic chamber, as discussed below.^85^

## Access to the Catalytic Chamber and Specificity for Alkanes

How alkanes navigate from the exterior to the active site of ACRs is an open exciting riddle. For methanogenic MCR, the future methane molecule enters the enzyme as methyl-S-CoM through the hydrophilic coenzyme cavity (Figure 1). It is accepted that the interaction of methyl-S-CoM favors the binding of HS-CoB through the same cavity,^58^ which triggers the reaction via a conformational switch, allowing heterodisulfide formation.^106^ Methane can be released simultaneously with the heterodisulfide. These enzymes can also generate ethane from ethyl-S-CoM and coenzyme B *in vitro*, albeit with lower rates, and therefore, the described process can be transposed to ethane.^107^ However, in the opposite reaction, the heterodisulfide will obstruct the coenzyme cavity. Therefore, methane must diffuse in the active site before the heterodisulfide binding or by another path. Xenon gassing experiments on methanogenic MCR crystals and bioinformatics predictions could not provide any reasonable hints for an existing gas channel for methanotrophic or methanogenic MCRs.^85,108^ This suggests that methane could access a heterodisulfide-loaded active site only by diffusion through the enzyme due to its relatively small size. However, as gassing experiments and simulations were performed on the inactive state, it is still possible that the active enzyme might have a different conformation that would allow diffusion of the gas.

**Figure 6:**
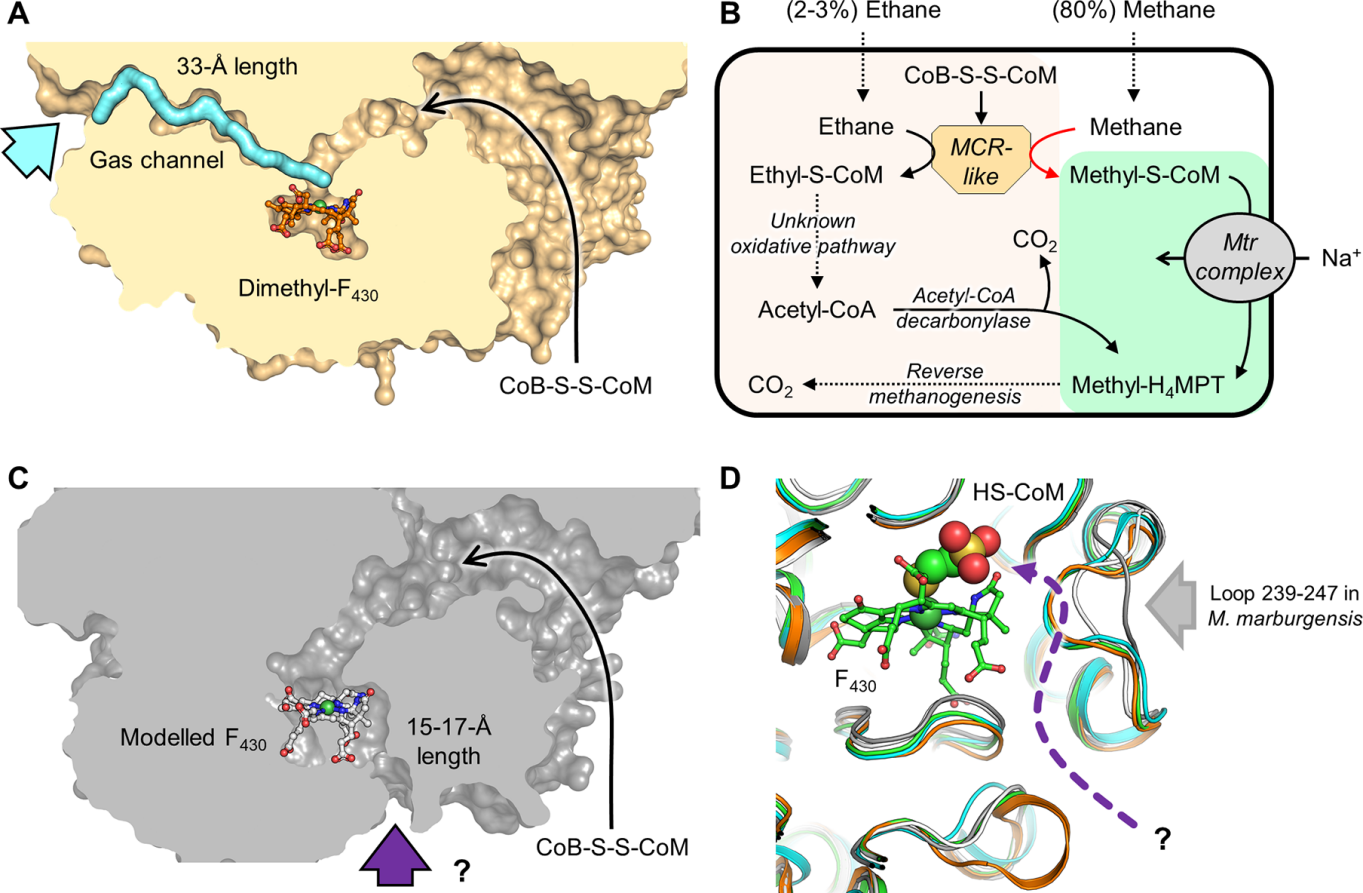
Access to the catalytic chamber. (A) Proposed ethane path to the active site in a cut-through view of the MCR homologue from *Ca*. E. thermophilum (PDB entry 7B1S). The protein is represented by an orange surface with its cofactor represented by balls and sticks. The experimentally demonstrated hydrophobic tunnel connecting the solvent to the active site is colored cyan with an arrow pointing to its entrance.^85^ (B) Pathway for ethane oxidation and the proposed proofreading role of the Mtr complex. The relative amounts of ethane and methane were taken from ref (8). (C) Proposed alkane path to the active site (entry highlighted by a purple arrow and a question mark) in the modeled structure of the MCR homologue of *Ca*. Methanoliparia (VUT24756.1, VUT24758.1, and VUT24757.1, cut-through view). The enzyme is represented by a gray surface with its cofactor represented by balls and sticks. (D) Superimposition of the structures of the methanogenic MCR (PDB entry 5A0Y, green), methanotrophic MCR (PDB entry 3SQG, cyan), the MCR homologue from *Ca*. E. thermophilum (PDB entry 7B1S, orange), and the modeled structures of the MCR homologues from *Ca*. Methanoliparia (gray) and *Ca.* S. butanivorans (OFV67021.1, OFV66073.1, and OFV66072.1, white). Proteins are represented by cartoons, and the F_430_ cofactor and HS-CoM (shown as spheres) from *M. marburgensis* MCR type I are shown to illustrate their expected positions. The putative alkane path is drawn as a purple dashed arrow with a question mark. In panels A, C, and D, oxygen, nitrogen, sulfur, and nickel are colored red, blue, yellow, and green, respectively.

In contrast, bioinformatics calculations and xenon gassing experiments confirmed the presence of a channel in the MCR homologue from *Ca*. E. thermophilum.^85^ The channel entry is formed by the insertions present only in ethanotrophs (Figures 2 and 3B), which protrude on the protein surface. The hydrophobic channel devoid of water molecules spans a distance of 33 Å and would facilitate the diffusion of ethane to the active site (Figure 6A). Its restrained geometry, tight diameter, and the modified residues located on the inner side (methylisoleucine, *N*^2^-methylhistidine, and methylcysteine) might act as a filter for alkane specificity and would explain why only ethyl-S-CoM can be detected upon addition of various short-chain alkanes in the culture.^53^ The natural environment of *Ca*. E. thermophilum contains a mixture of alkanes, and while a selective filter would explain the repulsion of molecules larger than ethane, methane (∼10 times more concentrated than ethane in the environment^109^) would still be able to enter and react. The accumulation of methyl-S-CoM in the cell would poison the ethanotrophic metabolism, and a proofreading system must exist. The methyl-H_4_MPT/S-CoM transferase (Mtr complex) encoded in the genomes of *Ca*. E. thermophilum and *Ca*. A. ethanivorans^41,53^ could be this metabolic corrector by turning the methyl-S-CoM into methyl-H_4_MPT, which can be metabolized through reverse methanogenesis (Figure 6B).

**Figure 7:**
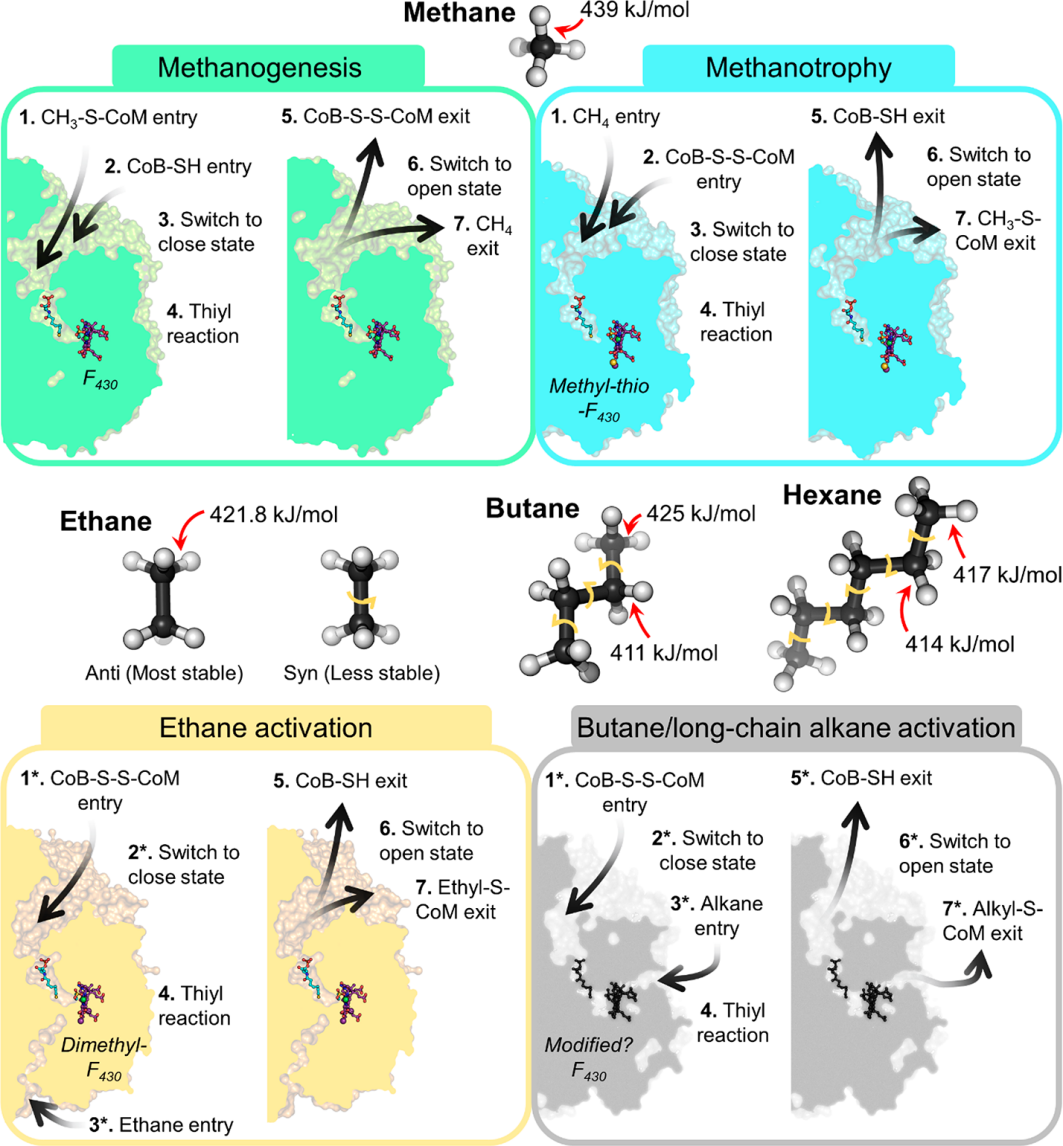
Bond dissociation energy between alkanes and differences in capture and release by ACRs. Alkanes (shown as balls and sticks) exhibit different C–H bond dissociation energies between the terminal and subterminal carbon.^24,113,133^ A proposed sequential process of alkane capture and release occurring in methanogenic (green), methanotrophic (cyan), ethane-activating (light orange), and long-chain alkane-activating (gray) ACRs is presented. Experimental structures (PDB entries 5A0Y, 3SQG, and 7B1S) or modeled structures (from *Ca*. Methanoliparia VUT24756.1, VUT24758.1, and VUT24757.1) are shown in a cut-through view with the cofactor and coenzymes shown as balls and sticks colored as in Figure 3. The unknown cofactor and coenzymes are colored black in the modeled structure. An asterisk indicates that both events can happen in reverse order of sequence.

On the basis of the MCR homologue sequence and molecular features of propane, butane, and long-chain alkanes, the presence of a hydrophobic channel comparable to that of the enzyme from ethanotrophs is disputable for several reasons. (1) The additional loops organizing the surface of the channel do not exist in homologues (Figure 2). (2) The required diameter for accommodating longer alkanes needs to be larger and might destabilize the overall protein architecture. (3) The minimum length of C13 alkanes consumed by *Ca*. Methanoliparia species is ∼15 Å (for a linear molecule), which is half of the distance of the channel observed in the *Ca.* E. thermophilum protein. (4) The diffusion rate of a longer alkane would be greatly impacted in such a long channel, and the polar head of the CoM adduct would impair its efficient removal. A shorter and wider hydrophobic cavity would be preferable due to the larger radius of the long-chain alkanes. As a rough approach, we used Alphafold2 to generate a model of the most expressed MCR from *Ca*. S. butanivorans [OFV67021.1, OFV66073.1, and OFV66072.1 (group d in Figure 2)] and an MCR homologue from *Ca*. Methanoliparia (VUT24756.1, VUT24758.1, and VUT24757.1). These models present no internal cavity that would be attributed to a gas channel. Instead, an open cleft can be observed in both models connecting the CoM-SH binding site to the solvent (Figure 6C). The cleft is caused by a single deletion at the predicted loop 239–246 (*M. marburgensis* numbering) observed in some MCR homologues in *Ca*. Syntrophoarchaeum, *Ca*. Methanoliparia, and *Ca*. Helarcheota/Lokiarchaeota (suggesting a role in mid- to long-chain alkane oxidation). In contrast, the methanogenic MCR of *Ca*. Methanoliparia has a classic loop (Figure 4). The models and their interpretation must be viewed with skepticism but allow tempting speculation about an open path that would favor the entry of bulky alkanes and efficient removal of alkyl-S-CoM (Figures 6C,D and 7). The cleft would span 15–17 Å in the modeled structures and would fit the size of a linear C13 alkane length. A longer alkane would protrude out of the cavity, regardless of its length or the group it could harbor (e.g., cyclohexyl), explaining the apparent wide substrate range of MCR homologues in *Ca*. Methanoliparia. Alternatively, long-chain alkane selectivity could occur by specific channel transporters at the membrane, hydrocarbon-carrier proteins, or microcompartments allowing an efficient and specific delivery to the MCR homologues.

The existence of this putative new path to the catalytic site would raise several issues. The F_430_ cofactor is sensitive to oxidation, which yields an inactive enzyme that must be reactivated by energy investment (as described below). A larger opening of the active site to the solvent would probably facilitate oxidation and therefore lead to energy losses. Moreover, the proposed localization of the long-chain alkane would induce a collision with the HS-CoM (Figure 6D). An adjustment of cofactor F_430_ or the coenzymes might allow an adequate position of the alkane facing the heterodisulfide. Hence, the existence of this putative long-chain alkane path might imply additional conformational changes in the enzyme, adding another level of complexity to the catalysis (Figure 7).

## Differences in Reaction Mechanisms?

The thiyl-radical mechanism proposed for methanogenic MCRs could theoretically be extended to all ACRs.^59,110^ The mechanism, already described in detail elsewhere,^47,59,110^ is briefly summarized. The heterodisulfide would bind the catalytic Ni in its active Ni^I^ state by the CoM sulfonate group.^110^ A transitory Ni–S interaction or long-range electron transfer would generate Ni^II^-S-CoM (or Ni^II^ and S^–^-CoM anion) and the CoB-S^●^ thiyl radical.^59,110^ The attack of this radical on the alkane will generate CoB-SH and an alkyl radical, the latter reacting with Ni^II^-S-CoM or the S^–^-CoM anion to form alkyl-S-CoM. The difference in the length of alkanes might, however, impact their reactivity.

The generation of methane from methyl-S-CoM and CoB-SH is an exergonic process with a Δ*G*° of approximately −30 kJ/mol of formed methane.^47^ The reverse reaction catalyzed by ANMEs is therefore largely unfavorable. To sustain an efficient methane-capture rate, an impressive amount of MCR is present in the cells to stimulate methane capture, and it has been suggested that methanotrophic MCRs could be as efficient as their methanogenic counterparts in fixing methane.^61,72,74,78,79,84,85^

Ethane should be in theory easier to activate with a Δ*G*° of ≈20 kJ/mol of fixed ethane,^47^ as reflected by the lower abundance of MCR in ethane oxidizers.^85^ The rotation of the carbon–carbon bonds offers a different landscape of conformation compared to that of the non-methane alkanes (Figure 7).^111,112^ Such different conformations differ in their stability and could also in theory differ in reactivity.^28^ It is therefore probable that MCR homologues using non-methane alkanes constrain a precise conformation to decrease the required activation energy. Unfortunately, the structure of the MCR homologue from *Ca*. E. thermophilum in an inactive state did not contain the reactive heterodisulfide and it is, therefore, difficult to draw any conclusions regarding an alkane (de)stabilization imposed by the protein environment.^85^

The dissociation energy of the H–C bond decreases with the length of the alkane (Figure 7). For *n*-alkanes ranging from C3 to C6, the H–C bond dissociation energy is higher for the terminal carbon than for the subterminal carbon, which makes the subterminal carbon easier to activate. It is probably for this reason that bacterial anaerobic alkane oxidizers activate their substrates on the subterminal carbon, even if activation of the terminal carbon has also been observed for propane.^18^ Accordingly, 1-butyl-S-CoM and 2-butyl-S-CoM (1:2 ratio) were detected upon the addition of butane in the butanotrophic consortium described by Laso-Pérez and colleagues.^52^ However, due to the presence of eight different MCR homologues in the culture, it is impossible to determine if the butyl-S-CoM mixture is formed by a single enzyme or multiple enzymes. Because 1-butyl-S-CoM would be the most plausible molecule used for the oxidative pathway, an unknown mutase was proposed to turn the 2-butyl-S-CoM into 1-butyl-S-CoM.^52^

With an increase in alkane length, the difference in activation energy between the terminal and subterminal carbon decreases and becomes negligible (Figure 7^113^). It would be therefore informative to decipher which carbon of the long-chain alkane is activated by the consortia dominated by *Ca*. Methanoliparia. A 1-alkyl-S-CoM adduct would be preferred on the basis of the proposed oxidative pathway.^25,54,55^

## Challenging Study of Nonmethanogenic MCRs

Modern sequencing methods allow the collection of a prodigious amount of genomic and metagenomics data from environmental samples and uncultivatable microorganisms. Consequently, the number of sequences encoding ACR homologues is constantly increasing, while this enzyme family remains poorly characterized. From the knowledge gathered about the few studied non-methanogenic MCRs,^84,85,100^ one could imagine that the different ACRs contain interesting structural features, post-translational modifications, cofactors, and coenzymes. However, technical limitations impair the study of these enzymes.

The current revolution of deep-machine learning such as Alphafold2 or RoseTTaFold allows the generation of accurate homology structural models from protein sequences.^114,115^ These new tools undoubtedly impact structure–function prediction and will be heavily used in the near future. Generating a library of ACR models from metagenome sequences would be a perfect solution for gaining structural insights and deriving hypotheses regarding their substrate specificities and reactivity, as we did above. But, the rising interrogation is how reliable these models are and if they could be sufficient to confirm hypotheses. The predicted models are dependent on the sequence (input), which may be wrong due to an incorrect initiating methionine, alternative stop codons, codon stop read-through, or variation of the genetic code. Furthermore, the post-translational modifications also interfere with structural predictions, as they drastically change the size and physicochemical properties of the corresponding residues (Figure 5^84,85,97,101,102^). The bulky F_430_ will also have to be considered during modeling as it influences the architecture of the active site (Figure 1C,D). The F_430_ itself can come in different flavors, which might affect its surroundings in an unpredictable manner, as for the nature and position of the coenzymes. Finally, ACRs are built of at least six chains, which complement each other by subtle contacts that are not always conserved even among close phyla.^101^ A prior analysis of residue, cofactor, and coenzyme modifications could be required to ensure correct modeling, even if subtle or yet uncharacterized modifications can easily be missed.^102^ For all of the reasons mentioned above, experimental structural characterization prevails to validate the models of these still poorly characterized enzymes.

The structural and biochemical characterization of MCR homologues from alkane oxidizers is limited by the available biomass, despite significant advances in the isolation and enrichment procedures.^116^ After years of cultivation, none of these archaeal alkanotrophs has been isolated, one of the main reasons being the dependency on other microorganisms. The use of artificial electron acceptors allows for the decoupling of the mutualistic archaeon–bacterium interaction and may lead to purer alkanotrophic cultures in the future.^61,117^ In the absence of a pure culture, protein purification must be performed from a complex microbial community. In some cases, the natural abundance of MCR in this protein mixture makes its purification still manageable even from the restricted heterogeneous biomass. The ultimate purification step via crystallization proved to be successful in the case of the Black Sea mats in which one isoform was sorted out from a mixture of five others.^84^ Structural characterization by X-ray crystallography is particularly well adapted for alkanotrophic MCR homologues.^84,85^ This approach, however, probably cannot be transposed to every available alkanotrophic enrichment due to the restrained yield and the complexity of microbes.

Protein overexpression in *Escherichia coli* is routinely used to produce large quantities of enzymes for structural and biochemical characterization and could be a good option for bypassing the biomass limitation of alkanotrophic cultures. In the case of ACRs, it should be theoretically feasible to obtain the active complex with co-expression of the whole F_430_ biosynthesis pathway,^118,119^ the enzymes responsible for the installation of some post-translational modifications,^98,99,104,105^ and the possible partners required for the activation and F_430_ insertion (e.g., *mcrC* and *mcrD* genes).^118,120,121^ An alternative and probably more adaptable setup is the production of ACRs in a methanogen, already successfully used for methanogenic and methanotrophic MCRs.^82,83,122^ Such a promising approach will, however, elude the installation of putative unknown post-translational modifications, and modified F_430_ cofactors or coenzymes will be overlooked. This could lead to biased interpretation, unlike the explorative approach with native organisms that has been used until now.

The scarcity of the enzymology data of MCRs may be striking. The reason lies in the highly reactive F_430_, which turns into an inactive state upon cell lysis.^47^ This inactive Ni^II^-silent state corresponds to most of the ACR deposited structures. Studies on methanogens revealed an ATP-dependent machinery responsible for the *in vivo* reactivation of the cofactor.^120^ Its conservation in alkanotrophic archaea has not yet been confirmed. Several alternative strategies have been employed to induce or preserve a methanogenic MCR active state: (1) gassing cells with CO_2_-free H_2_ prior to harvesting to artificially decrease the CoM-S-S-CoB concentration and keep the enzyme in a reduced active state,^123^ (2) addition of 20 mM sulfide to the culture media prior to harvesting,^124^ (3) *in vitro* reactivation with titanium citrate of the pure enzyme extracted from cells gassed with N_2_/CO_2_ (80/20) before harvesting,^125^ and (4) addition of carbon monoxide to the cell extract.^126^ All of these methods are based on the reduction of MCR and/or coenzymes by chemicals or enzymatic reactions. Gassing with H_2_ probably cannot be used in alkanotrophs because most of them are not expressing hydrogenases and therefore are probably not coupling the heterodisulfide reduction with H_2_ oxidation unlike hydrogenotrophic methanogens.^120^ The presence of sulfide in the medium is also probably not sufficient because the structurally characterized nonmethanogenic MCRs were extracted from organisms living with relatively high sulfide concentrations and still exhibit an inactive state.^84,85^ However, the CO-gassing strategy could be considered because many alkanotrophs are supposed to be dependent on a CO-dehydrogenase belonging to the acetyl-CoA decarbonylase complex (Figure 6B^25,41,52−55^). Monitoring alkane consumption *in vitro* with a purified enzyme maintained in the active state would finally provide the experimental validation of alkane activation by a nonmethanogenic MCR.

## Conclusions and Future Perspectives

The MCR is the second, if not the first, most abundant enzyme on Earth.^47^ It catalyzes the methane production or its capture, a crucial step in the metabolism of numerous archaea occurring in anaerobic ecological niches. The enzyme has been studied for decades, unraveling its overall structure, cofactor, coenzymes, post-translational modifications, and key steps of the reaction mechanism. However, some gaps in our knowledge of these enzymes remain.

The reaction process has still not been fully characterized, and some proposed intermediates in the catalytic cycle remain to be experimentally verified.^110^ Almost all of the work on the enzyme reaction mechanism has been performed on a single enzyme (MCR type I from *M. marburgensis*), and if the structural characterization of these enzymes were performed in a wider range of organisms (see Figure 2), that would represent only a restrained part of the known methanogenic and methanotrophic archaea. Furthermore, the structures obtained describe an MCR in an inactive state, which is not representative of any step of the proposed catalytic mechanism. Despite the description of the biological reactivation system^120^ and of several methods allowing the purification of an active enzyme,^123−126^ structural data of the active enzyme need to be imperatively unveiled to obtain the whole catalytic picture.

The inactivation of the enzyme and the difficulty of its heterologous expression in genetically tractable systems in methanogenic archaea slowed the functional elucidation of the post-translationally modified residues. While recent investigations provided new insights, the physiological role of each post-translational modification and the reason for their (non)conservation are still unclear.

Studies on anaerobic alkane oxidizers expanded new horizons on the functional diversity of MCR homologues. The picture of the enzyme’s distribution in the *Archaea* superkingdom is only beginning to sharpen, and more insights regarding its evolution will be obtained from future metagenomics data and phylogeny analyses. We now need to (re)evaluate the width of the contribution and impact of ACRs on the anaerobic hydrocarbon oxidation compared to bacterial systems [e.g., alkylsuccinate synthase, Ass/Mas system (see above)]. Compared to the latter, ACRs appear to have a wider substrate panel, ranging from C1 to C22 (even nonlinear), and the limit of the ACR family regarding their potential substrates is still unknown. The reaction with a longer alkane implies novel structural and mechanistic features to allow efficient substrate access to the active site and product removal, which diversifies and complicates the reaction mechanism of this enzyme class.

In theory, an organism producing all different variations of ACRs could be a generalist alkanotroph with the ability to oxidize all alkanes available in its environment. Such a generalist has not yet been identified or suggested, probably because the specialized downstream metabolism requires the utilization of a defined alkyl-S-CoM. Indeed, the unknown steps occurring in the conversion of the alkyl-S-CoM to acyl-S-CoA could explain the apparent necessity for alkanotrophs to specialize in the use of a precise alkane to avoid a scrambling of its central metabolism. Such steps are uncharted chemistry, and new enzymes with singular reactions await discovery. Further studies must be carried out to decipher this process if we want to draw a complete image of the archaeal alkanotrophic pathways.
